# Identification immune response genes in psoriasis after treatment with secukinumab

**DOI:** 10.1186/s12920-023-01507-w

**Published:** 2023-04-07

**Authors:** Jing Wang, Yufang Liu, Yuxin Zhang, Shiyan Wang, Shaomei Kang, Ningyu Mi, Ruxin Li, Yulin Zou

**Affiliations:** 1 Department of Dermatology, Renmin Hospital, Hubei University of Medicine, Shiyan, 442000 Hubei People’s Republic of China; 2 Department of Dermatology, Jinzhou Medical University Graduate Training Base, Renmin Hospital, Hubei University of Medicine, Shiyan, 442000 Hubei People’s Republic of China

**Keywords:** Severe psoriasis, Secukinumab, Immune response, PPI

## Abstract

**Background:**

Secukinumab is a fully human IgG1κ MoAb that selectively binds to IL-17A with high affinity, and it has been proven effective for the treatment of psoriasis. However, the immune response pathways and mechanisms during the treatment are still masked. Therefore, the current study was designed to investigate the potential immune response genes via bioinformatics approaches.

**Methods:**

Gene expression data of severe plaque-type psoriasis was retrieved from the GEO database. Quantification of immune infiltration by ssGSEA and identification of differentially infiltrated immune cells were conducted to validate the treatment effect of secukinumab. After data processing, differentially expressed genes were identified between the treatment and untreated group. TC-seq was employed to analyze the trend of gene expression and clustering analysis. IL-17 therapeutic immune response genes were selected by taking the intersection of the genes inside the key cluster set and the MAD3-PSO geneset. Based on these therapeutic response genes, protein–protein interaction networks were built for key hub gene selection. These hub genes would work as potential immune response genes, and be validated via an external dataset.

**Results:**

Enrichment scores calculated by ssGSEA illustrated that the immune infiltration level of T cells had a strong difference before and after medication, which validated the treatment effect of Secukinumab. 1525 genes that have significantly different expression patterns before and after treatment were extracted for further analysis, and the enrichment result shows that these genes have the function related to epidermal development, differentiation, and keratinocytes differentiation. After overlapping candidate genes with MAD3-PSO gene set, 695 genes were defined as anti-IL7A treatment immune response genes, which were mainly enriched in receptor signaling and IL-17 signaling pathways. Hub gene were pinpointed from the PPI network constructed by anti-IL7A treatment immune response genes, their expression pattern fits TC-seq gene expression pattern.

**Conclusion:**

Our study revealed the potential anti-IL7A treatment immune response genes, and the central hub genes, which may act critical roles in Secukinumab, induced immune response. This would open up a novel and effective avenue for the treatment of psoriasis.

## Introduction

Secukinumab, a first in class, anti-IL-17A monoclonal antibody, fully human-has demonstrated sustained and strong efficacy with a fine safety profile, in the treatment of patients with psoriatic arthritis, ankylosing spondylitis and moderate-to-severe plaque psoriasis [1–6]. Psoriasis is an inflammatory, chronic disease of the nails and skin with a popularity that varies with ethnicity [7]. The worldwide prevalence is nearly 2%, but varies according to different regions [8]. In addition to influencing the joints and skins, it has been associated with an increased risk for many comorbidities, such as cardiovascular disease, it can also have a negative impact on patients’ psychosocial welfare [9]. T cell activation, which associated with the secretion of proinflammatory cytokines drive the psoriasis, including interleukin (IL)-17A, tumor necrosis factor-α(TNF-α), interferon IFN-γ and IL-22 [10, 11]. Data from clinical and in vitro studies indicate that IL-17A, principally drives varies within affected tissues, a critical effector cytokine in the IL-23/IL-17 immunologic pathway [12–16]. Interleukin (IL)-17A induces the expression of keratinocyte-derived products, for instance, anti-microbial peptides, chemokines, and cytokines, creating feed-forward loops that amplify and sustain skin inflammation [17, 18].

Secukinumab was approved in 2015 for treatment of patients with psoriasis as the first IL-17A inhibitor, and nearly one year later it was also approved for PsA. As a a fully human anti-IL-17A IgG1 monoclonal antibody, it can neutralize and selectively bind IL-17A. As we all know six members, IL-17A-F, compose the IL-17 cytokine family. Both IL-17F and IL-17A are secreted by Th17-cells, and other immune cells. IL-17A is about 10–30-fold more potent than Interleukin-17F [16]. In phase III studies about Secukinumab, the ratio of patients who achieved PASI75 was 75.9–86.7% at week 12 with secukinumab 300 mg (administered once weekly for four weeks starting at week zero, then every four weeks) and 0–4.9% with placebo [1–3]. According to head-to-head studies, secukinumab to be superior compared with both ustekinumab and etanercept [19, 20]. The ratios of patients (secukinumab vs ustekinumab) with a PASI90 response were 76% vs 61% and that with a PASI100 response were 46% vs 36% after 52 weeks of treatment [19]. Conclusively, secukinumab is highly effective, and compared with ustekinumab, it has has a faster onset of action and higher PASI90—and PASI100 response rates.

Although secukinumab has been shown to be well effective and tolerated in treating patients with PsA and plaque psoriasis in multiple clinical trials [3, 19, 21, 22] with up to 5-year follow up [23]. But so far, the specific target and immune response mechanism are not clear. Therefore, this study used transcriptome analysis to explore the potential immune response genes of secukinumab, so as to provide guidance for the further development of more efficient antibodies.

## Materials and methods

### Data source

In the current study, gene expression profiles of the skin of psoriatic patients punch in GSE137218 were downloaded from the gene expression omnibus (GEO) database. The tissue extracted for RNA microarray transcriptomic analysis is the skin punch biopsies, which were taken from nonlesional (NL) and lesional (LS) psoriatic skin of psoriatic patients before secukinumab treatment and from lesional psoriatic skin at day 4, 14, 42 and 84 during secukinumab treatment.

### Quantification of immune infiltration and identification of differential immune cells

The immune infiltration levels were quantified using enrichment scores calculated by ssGSEA [24]. 28 kinds of immune cell gene sets are calculated, and the result represents the degree to which specific immune cell gene sets are up-regulated or down-regulated in the sample.

### Identification and functional enrichment analysis of DEGs

In this study, Limma [25] R package was used to identify differentially expressed genes among secukinumab-untreated psoriatic group (Day 0), secukinumab-treated psoriatic groups (Day 4, Day 14, Day 42 and Day 84) and healthy control group (NC). The screening criteria are as follows: Log2FC value is greater than 1.5, and p-value is less than or equal to 0.05. Then the intersected differentially expressed genes found in Day 0 VS Day 4, Day 0 VS Day 14, Day 0 VS Day 42, Day 0 VS Day 84, and Day 0 VS NC were extracted and defined as DEGs in this study. R package clusterprofiler [26] was applied for GO, KEGG, and Reactome pathway enrichment analyses of selected DEGs and further enrichment analysis mentioned following.

### Anti-IL17A immuue response gene identification

TC-seq [27], Quantitative and differential analysis of epigenomic and transcriptomic time course sequencing data, clustering analysis and visualization of temporal patterns of time course data, can be applied to cluster genes based on gene expression patterns. In this study, TC-seq was employed for clustering the DEGs mentioned in 2.3. The genes included in the central clusters were extracted as candidate genes. The intersection between candidate genes and MAD3-PSO [28] gene sets are defined as anti-IL7A immune response genes.

### Construction of protein–protein network and hub gene selection

The anti-IL7A immune response genes were defined as up-regulated genes and down-regulate genes based on their expression pattern in previous TC-seq clusters. For the construction of the protein–protein interaction network, anti-IL7A immune response genes were input into the String database, then generated the up-regulated gene PPI and down-regulated gene PPI. The protein–protein interaction network was constructed and visualized by Cytoscape software. MCODE plug-in was used to minning the core modules in PPI networks. Thresholds were set as connectivity > 2, Node Score > 0.2, K-score > 2, and maximum depth > 100, then the top1 modules were selected as key modules, and the genes contained in the modules were the key Hub genes. Their expression pattern were validated by TC-seq and GSE158448.

### Statistical analysis

All data were analyzed with R software (version 4.0.0). Wilcoxon test was used to compare the data between two groups, and significant difference was considered as *p*-value < 0.05 (Table 1).

**Table 1:** R package

Software	Version	Usage
Limma	3.4.8	Differential expressed gene analysis
Tcseq	1.1.6	Gene expression pattern cluster
String	1.4	Protein–protein interaction network
Gsva	1.4	ssGSEA analysis
clusterprofiler	4.0	Enrichment analysis

## Results

### Immune infiltration levels shows the treatment effect of secukinmab

**Fig. 1:**
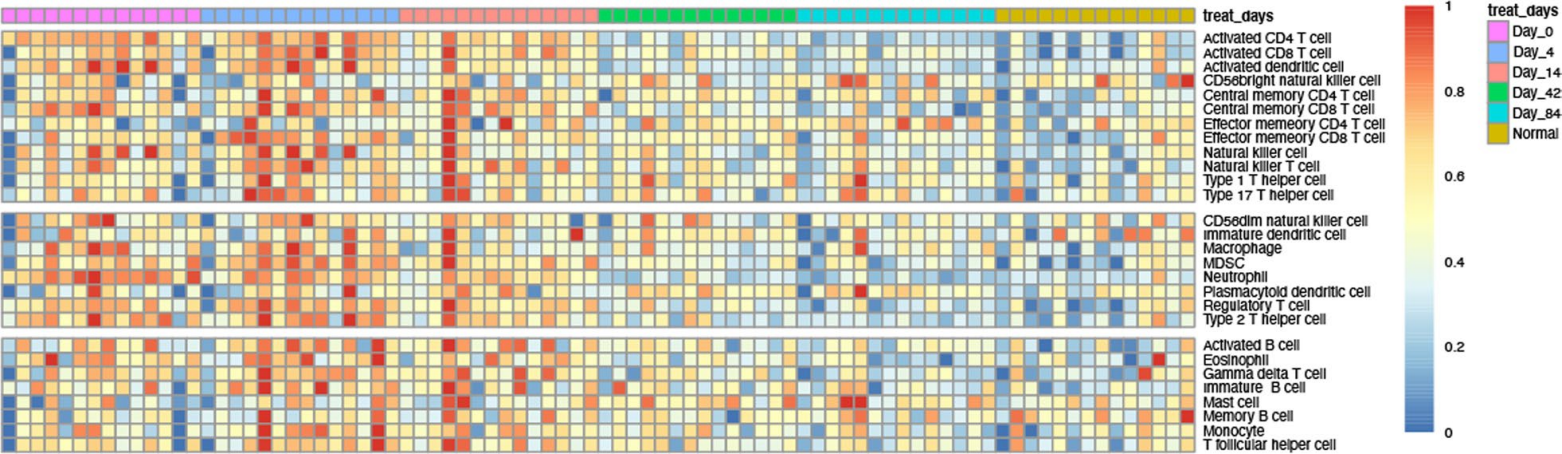
ssGSEA immune infiltration enrichment score heatmap

**Fig. 2:**
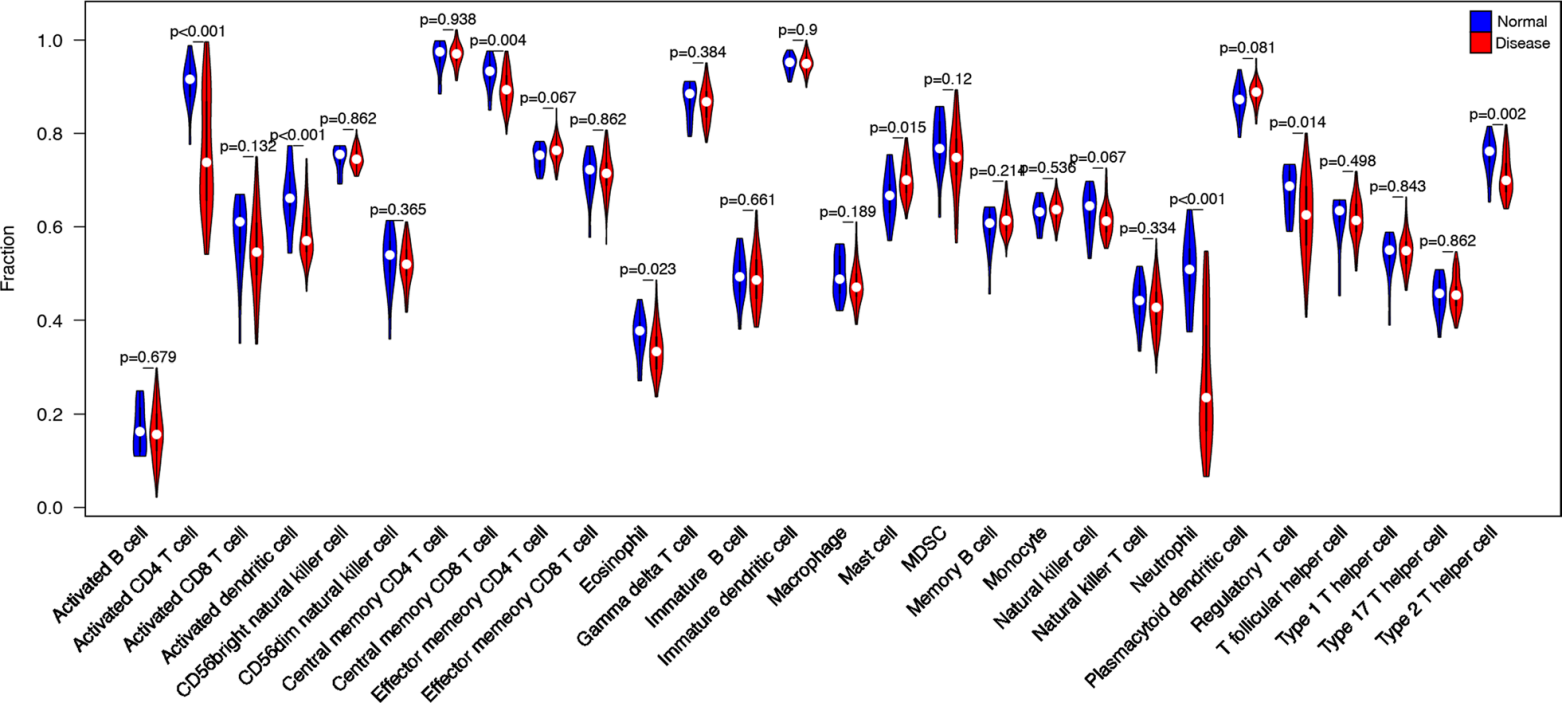
Day 0 group vs other groups immune-related gene expression differences

By the ssGSEA method, the infiltration of 28 immune cells was analyzed that a higher ssGSEA score indicated more infiltrated immune cells. We found that the immune infiltration level of T cells shows a strong difference between Day 0 and Day 84, illustrating the treatment effect of secukinumab in psoriasis (Fig. 1 ssGSEA immune infiltration enrichment score heatmap). Subsequently, the mean immune infiltration score of each sample in Day 4, Day 14, Day 42, Day 84 and NC groups were calculated and compared with Day 0. It can be observed that activated CD4 T cell, activated dendritic cell central memory CD8 T cell, eosinophil mast cell, neutrophil, and type 2 T helper cell have significant differences (*P* < 0.05) between Day 0 groups and other groups. (Fig. 2 Day 0 group vs other groups immune-related gene expression differences).

### The expressions of candidate anti-IL17A immune response gene shows gradually down regulation with treatment duration

**Fig. 3:**
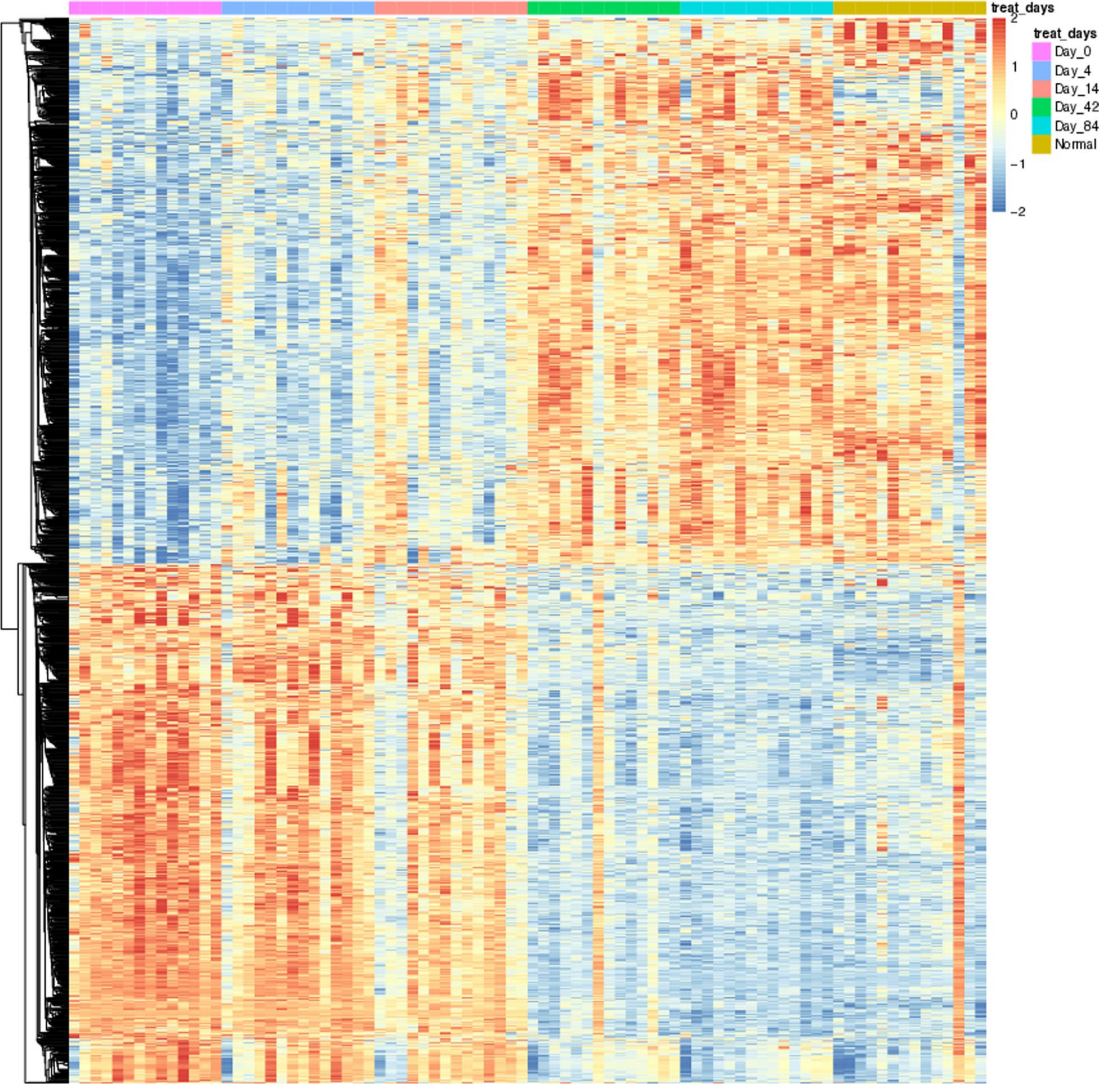
DEGs expression heatmap

**Fig. 4:**
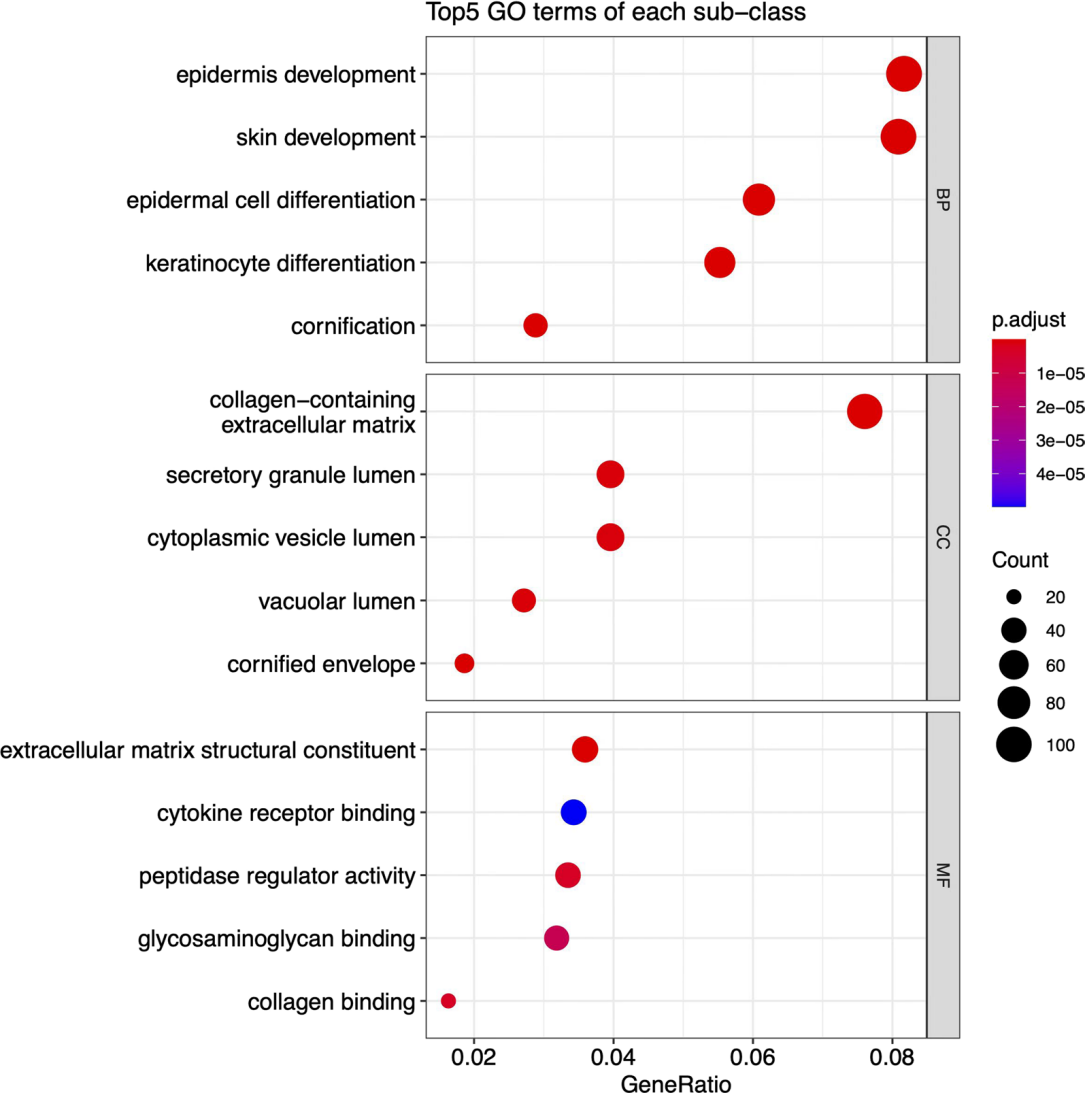
GO function enrichment

**Fig. 5:**
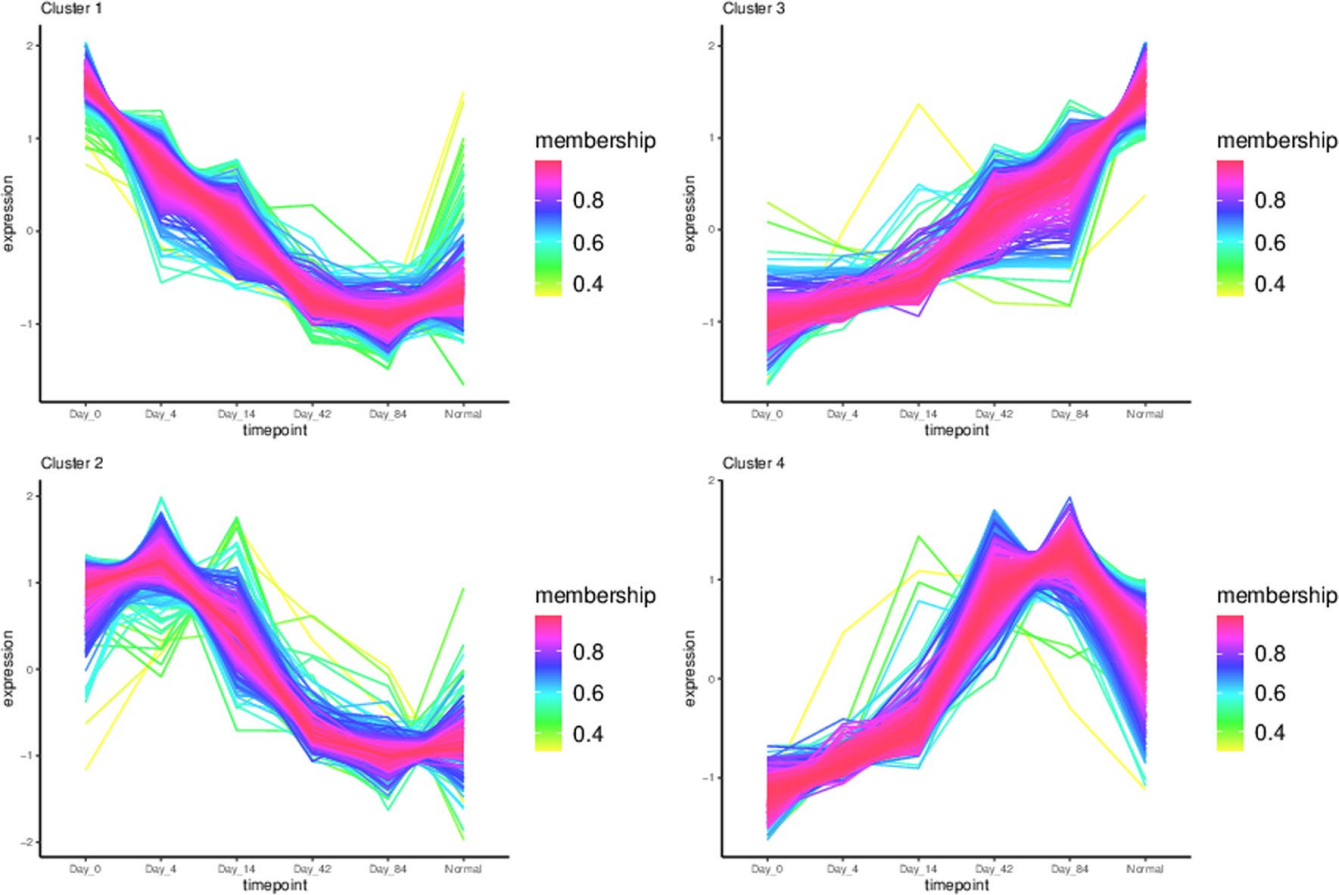
Gene expression pattern cluster

**Fig. 6:**
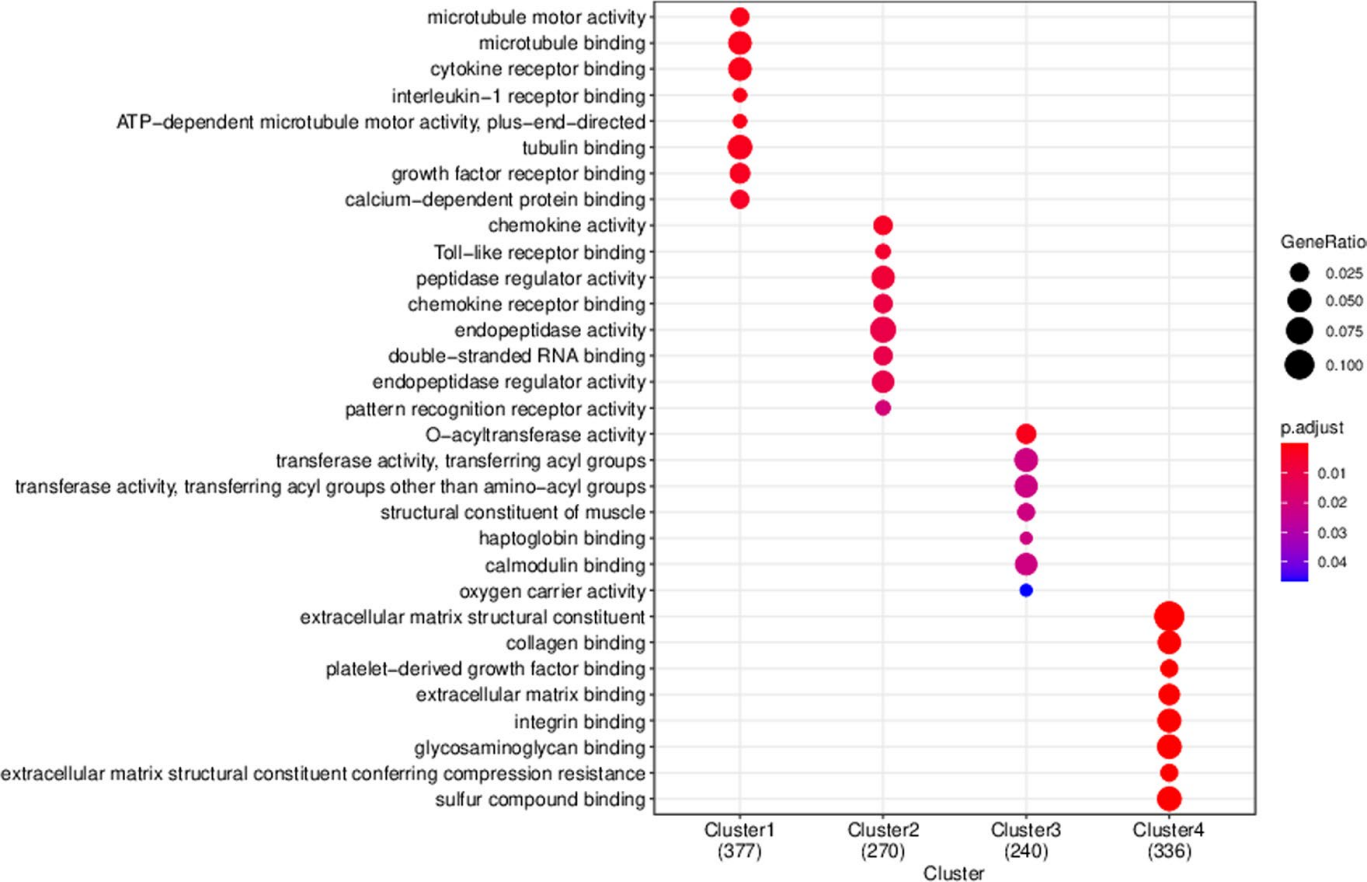
GO function enrichment

To initially investigate the intrinsic differences in transcriptomic before and after secukinumab treatment, we performed differential expressed gene analysis between different drug treatment duration and control, and disease samples vs control samples. We obtained a total of 1936 DEGs (Fig. 3 DEGs expression heatmap), Functional enrichment analysis revealed that the DEGs were mainly related to epidermis cell development and differentiation, skin development, keratinocyte differentiation, which are consistent with the physiopathologic mechanism of psoriasis. (Fig. 4 GO function enrichment) TC-seq clustered the DEGs as 4 distinct clusters, in which genes in cluster1 and 2 were downregulated and genes in clusters 3 and 4 were up-regulated. **(**Fig. 5 Gene expression pattern cluster), All 1525 genes present in these 4 clusters are extracted as candidate genes. Gene ontology function enrichment analysis of these candidate genes showed that they were significantly enriched into receptor signaling and receptor binding. (Fig. 6 GO function enrichment).

### Identification of anti-IL17A immune response gene

**Fig. 7:**
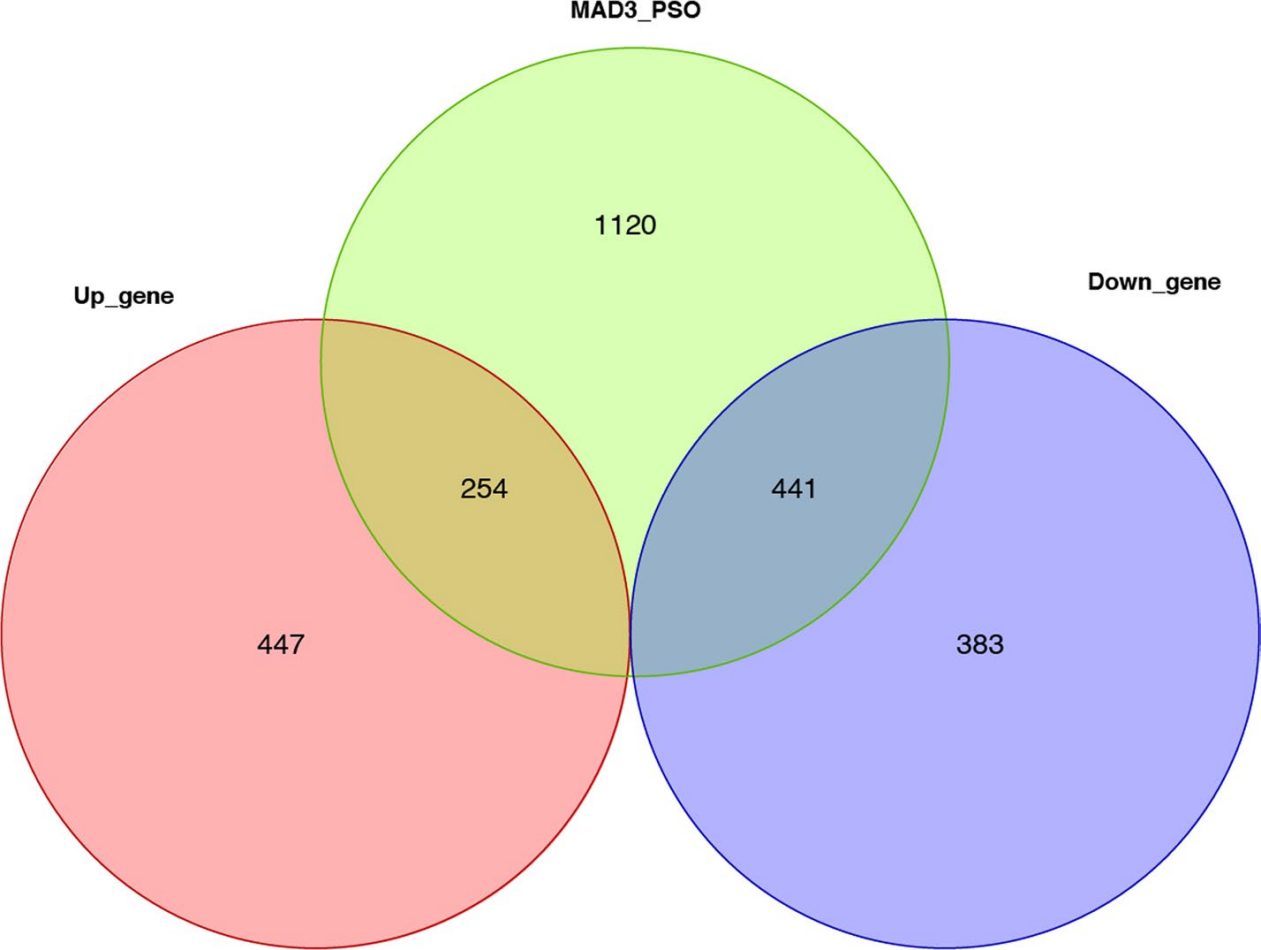
Intersection gene venn

**Fig. 8:**
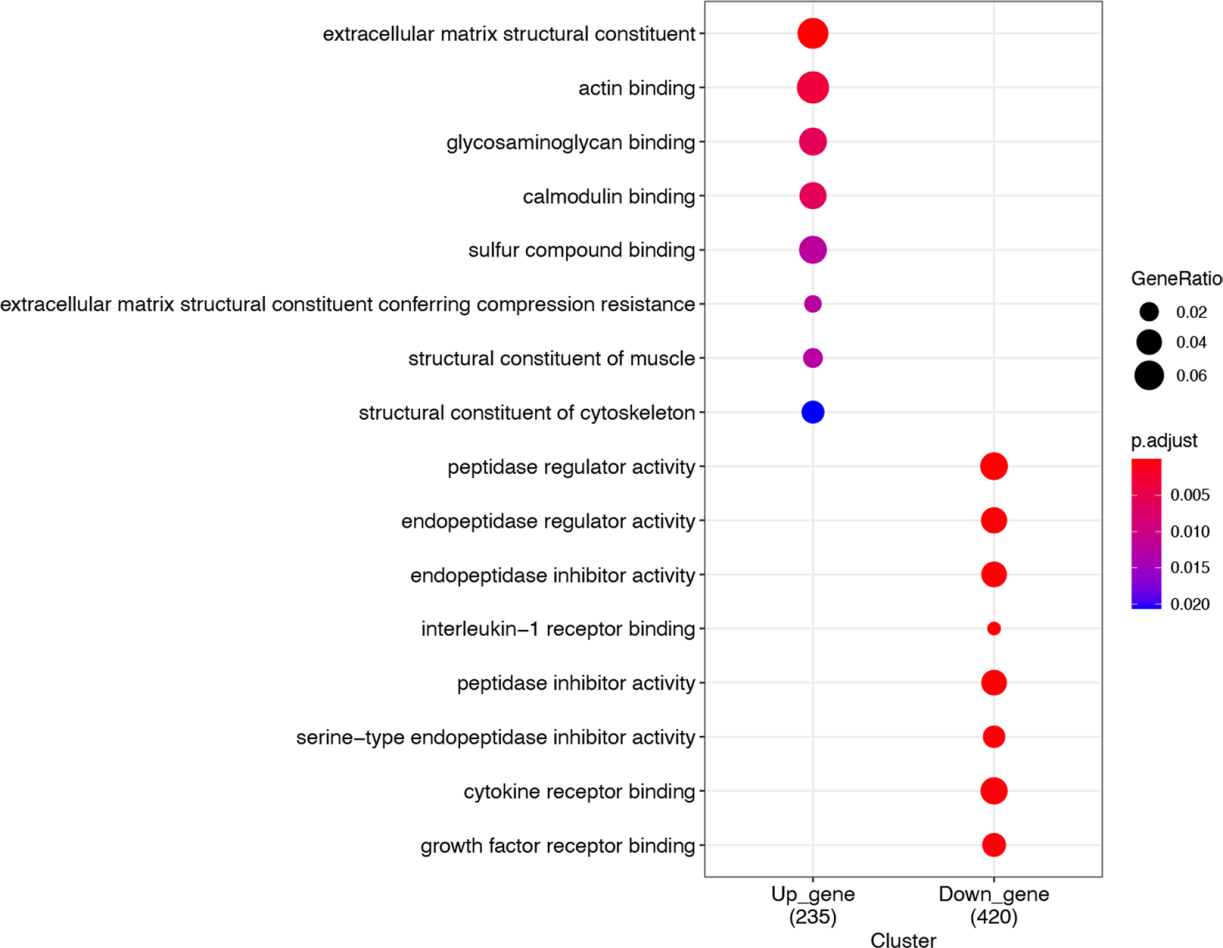
GO function enrichment

**Fig. 9:**
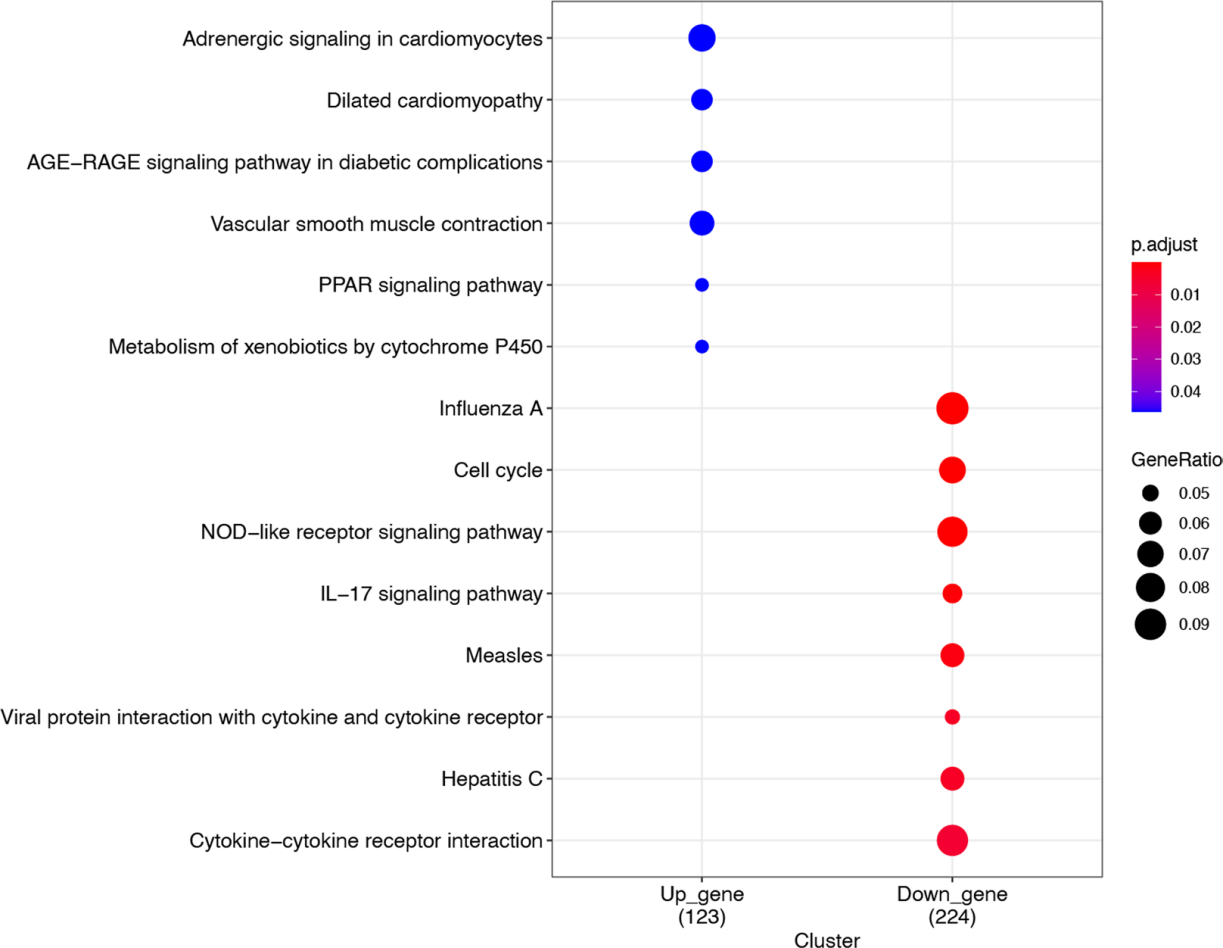
KEGG pathway enrichment

**Fig. 10:**
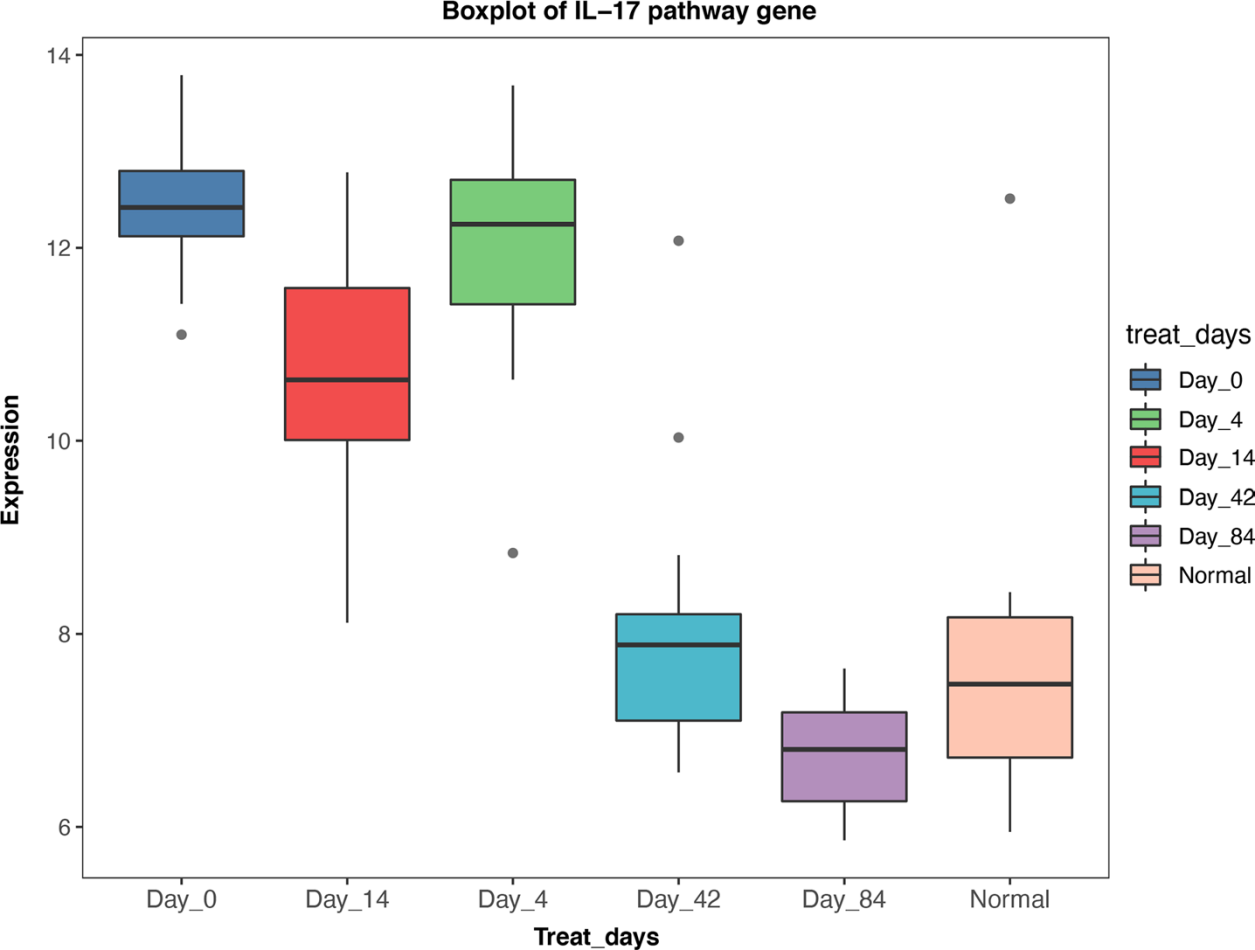
IL-17 pathway-related gene expression

Given the intersection of the candidate genes with the MAD3-PSPO gene set, 650 genes are obtained and defined as anti-IL17A immune response genes (Fig. 7 Intersection gene venn). GO and KEGG enrichment analysis predicts that the gene function enrichment on DNA replication, apoptosis, and signal pathways regulating functions, such as infectious related regulation and IL-17 receptor signal pathway (Fig. 8 GO function enrichment Fig. 9 KEGG pathway enrichment). Specifically, the expression pattern of IL-17 pathways related gene gradually down-regulated with the duration of the treatment (Fig. 10 IL-17 pathway-related gene expression).

### PPI network analysis and hub genes

**Fig. 11:**
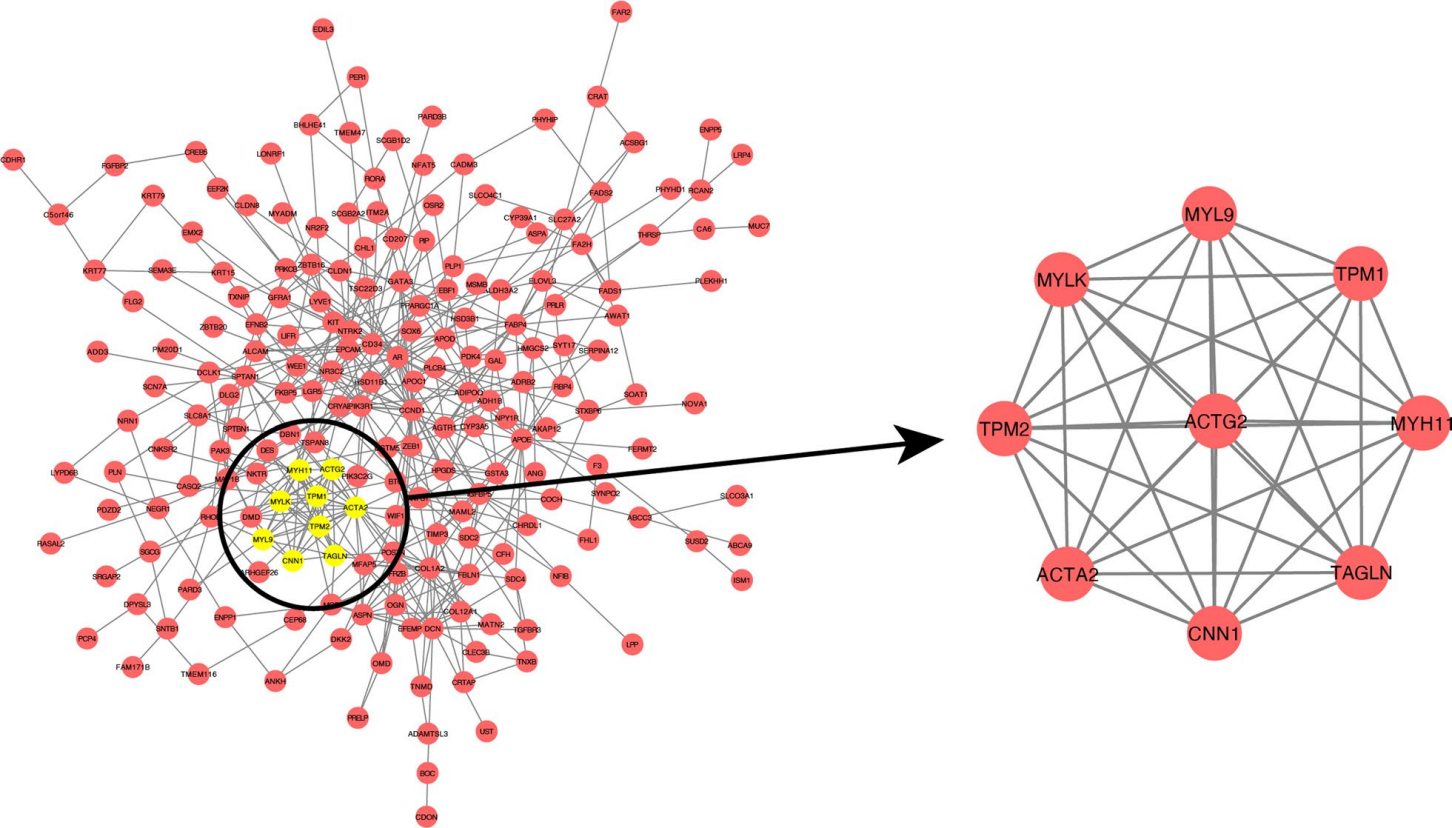
Up_regulated gene PPI protein interaction network

**Fig. 12:**
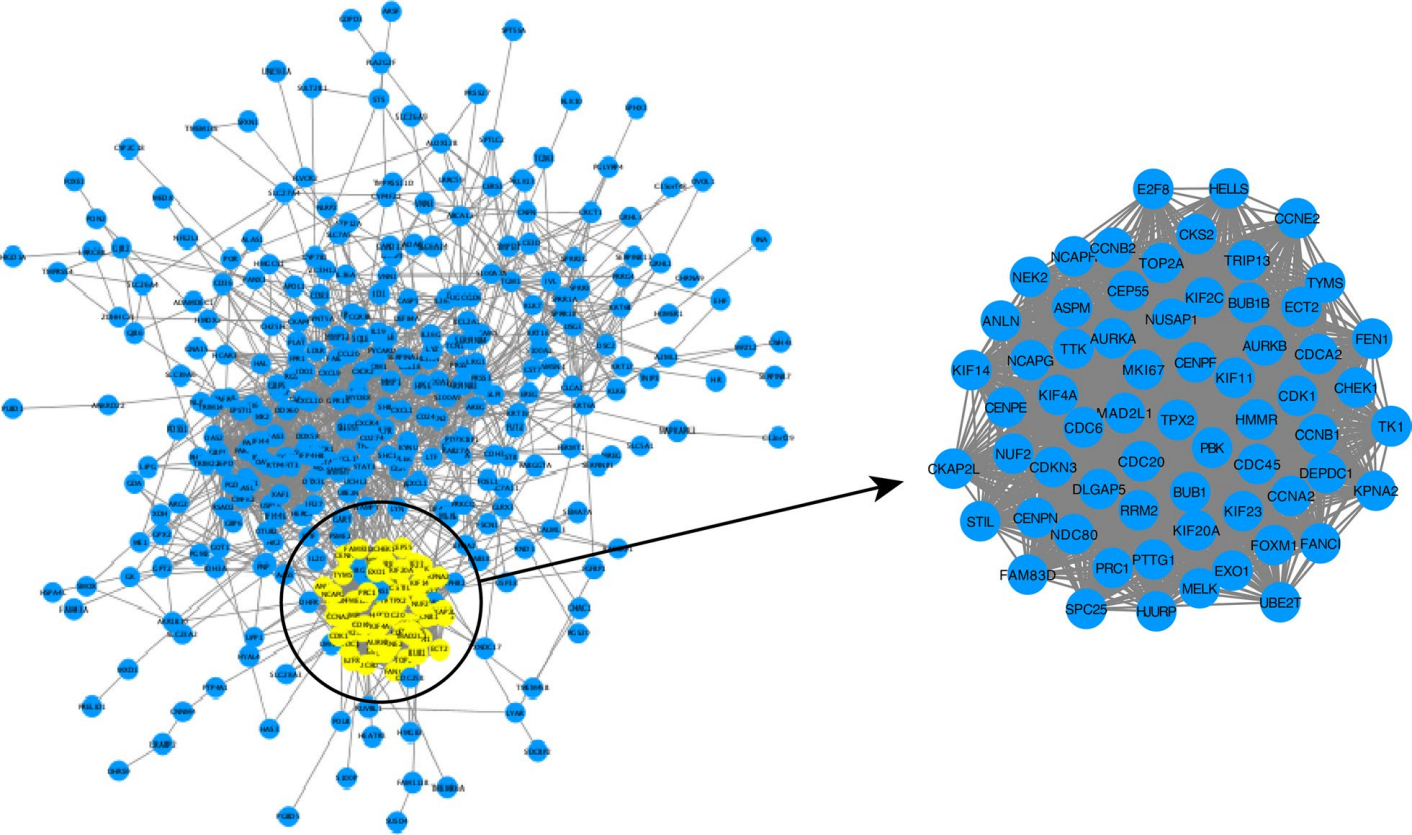
Down_regulated gene PPI protein interaction network

**Fig. 13:**
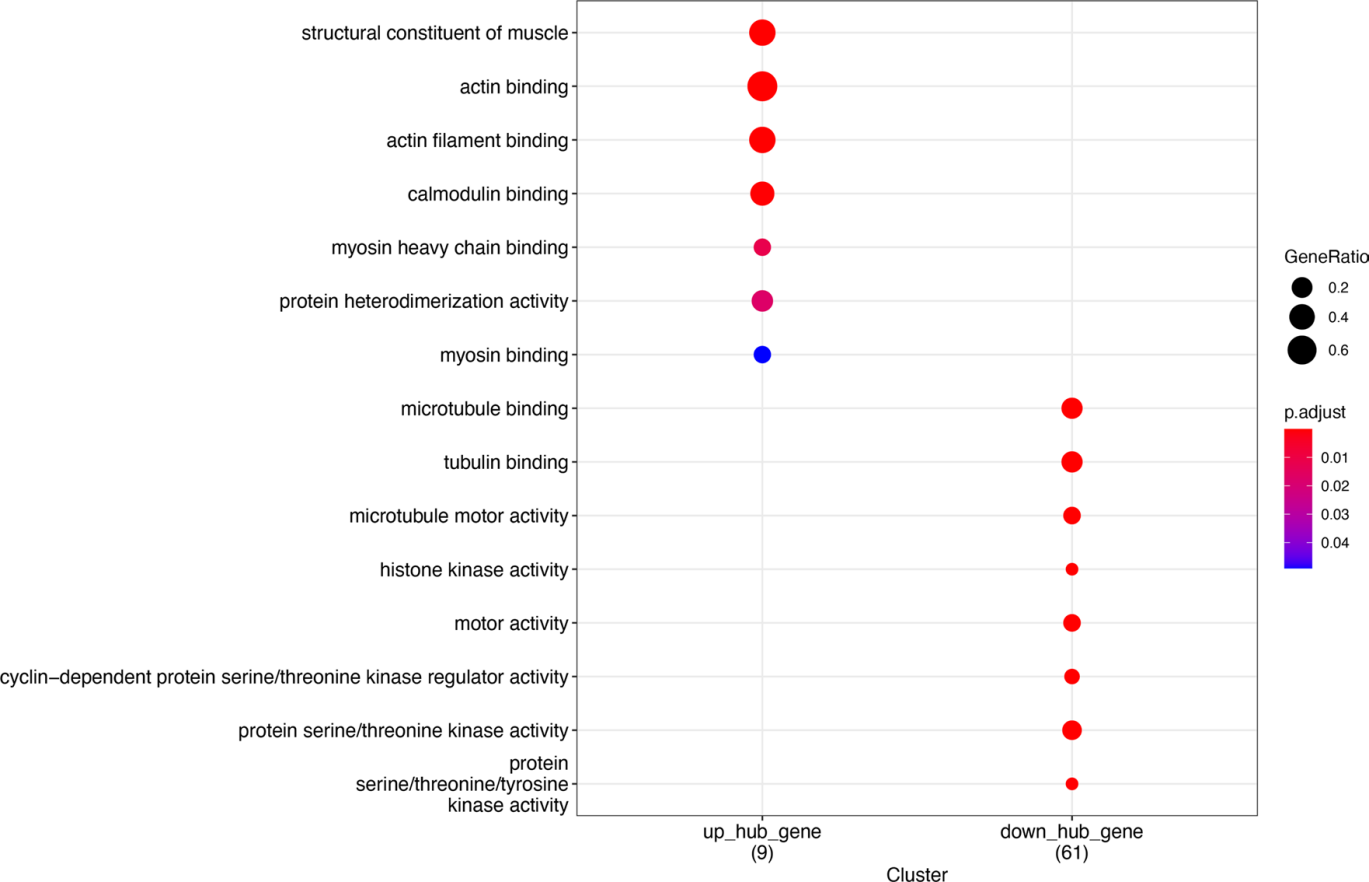
Hub gene GO function enrichment

**Fig. 14:**
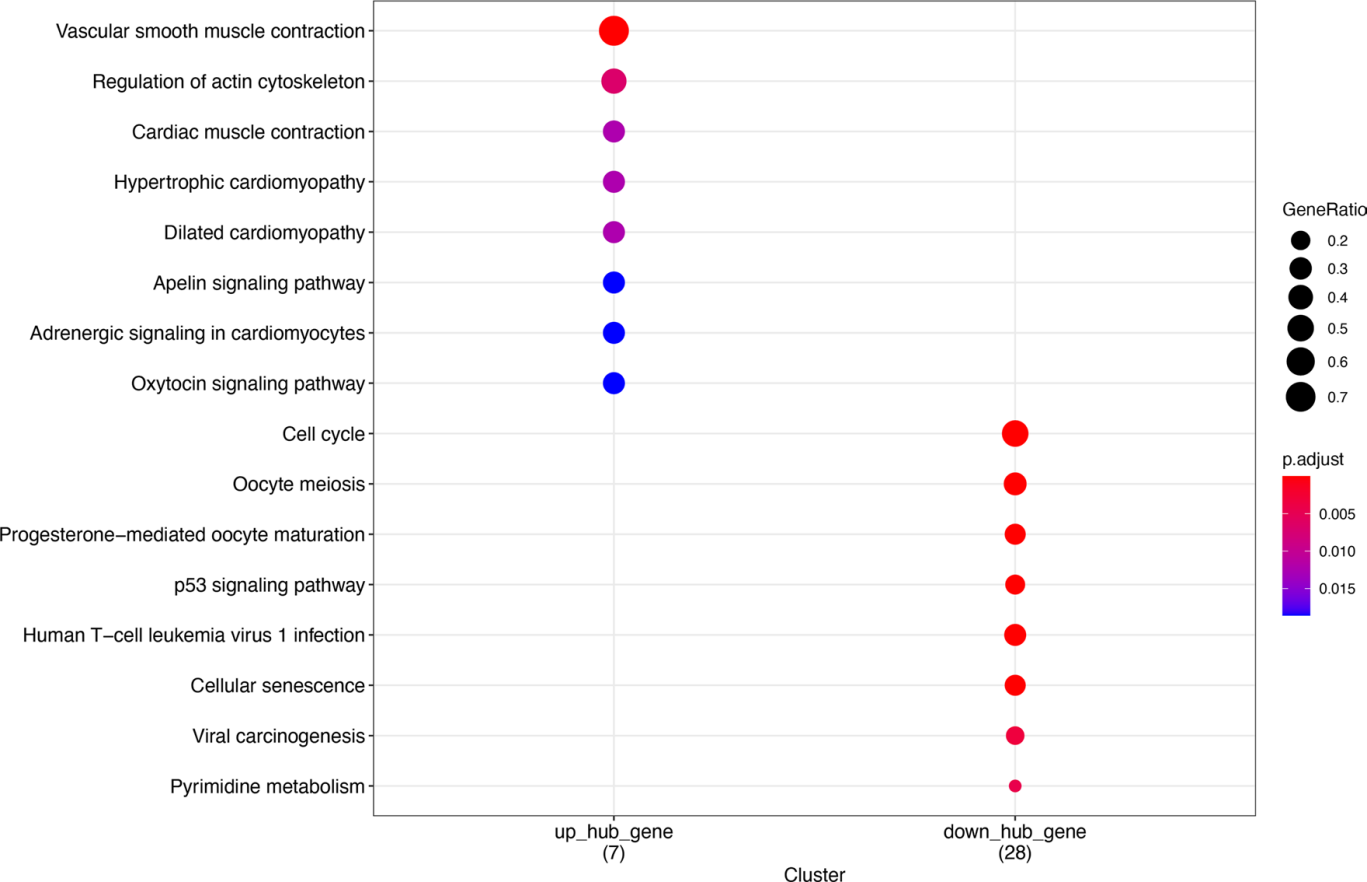
Hub gene KEGG pathway enrichment

To identify hub genes in the anti-IL17A immune response genes, up regulation genes-genes in cluster 1 and 2, and downregulation gene-genes in cluster 3 and 4, were used to construct PPI network. Among them, the prior model (model 1) are selected as hub model in two PPI respectively (Fig. 11 Up_regulated gene PPI protein interaction network Fig. 12 Down_regulated gene PPI protein interaction network). 73 of them come from the down-regulated gene PPI network and 9 of them are from the down-regulated gene PPI network. Functional enrichment analysis of hug genes revealed that they may have the function of actin-binding and calmodulin-binding. The expression pattern of these hub genes shows strong difference between treatment group (day 0, 4, 14, 42, 84) and control group, illustrate that the difference is consist with the TC-seq gene expression pattern. (Fig. 13 Hub gene GO function enrichment Fig. 14 Hub gene KEGG pathway enrichment).

**Fig. 15:**
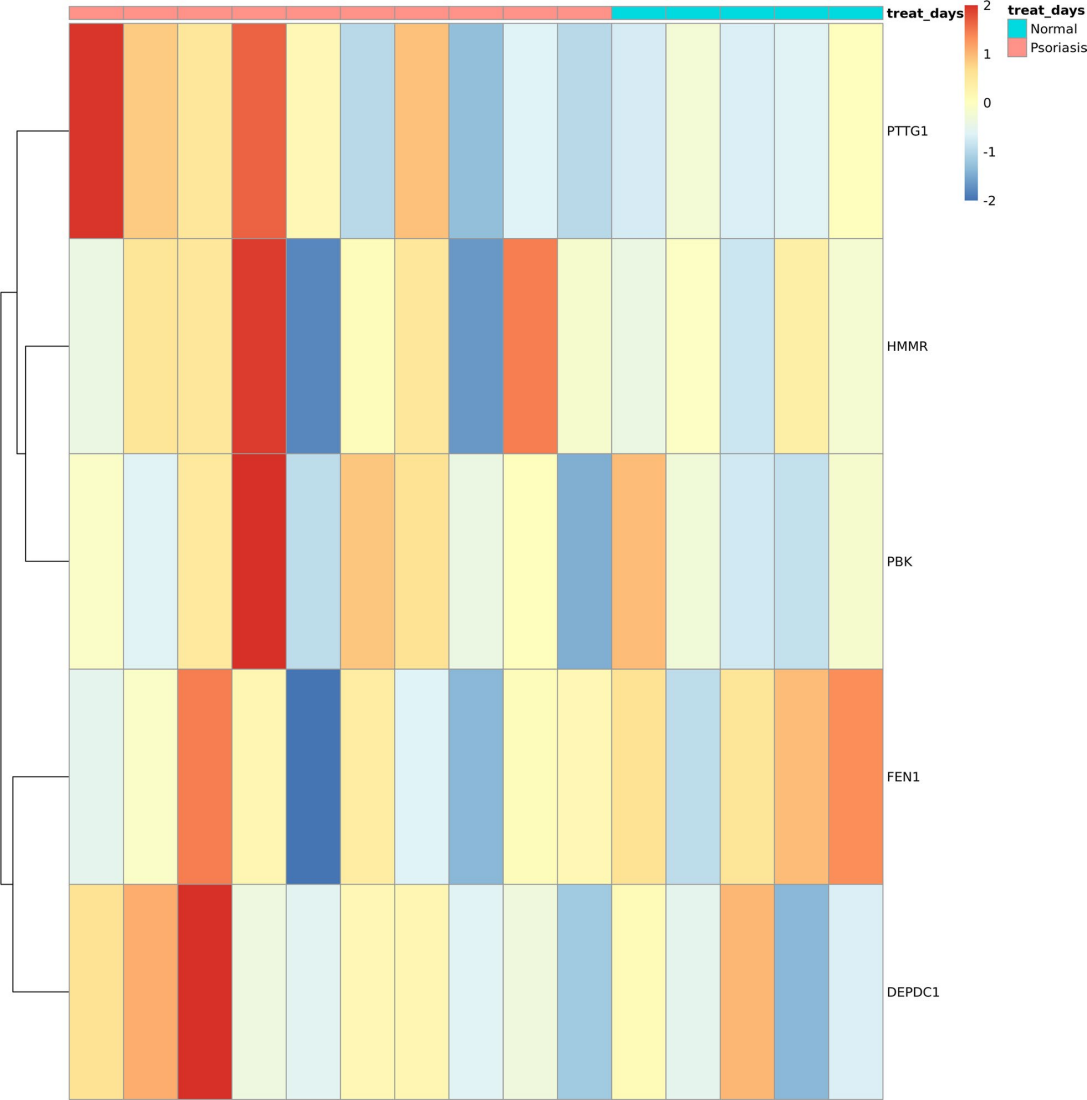
Up_regulated hub gene differential expression heatmap

**Fig. 16:**
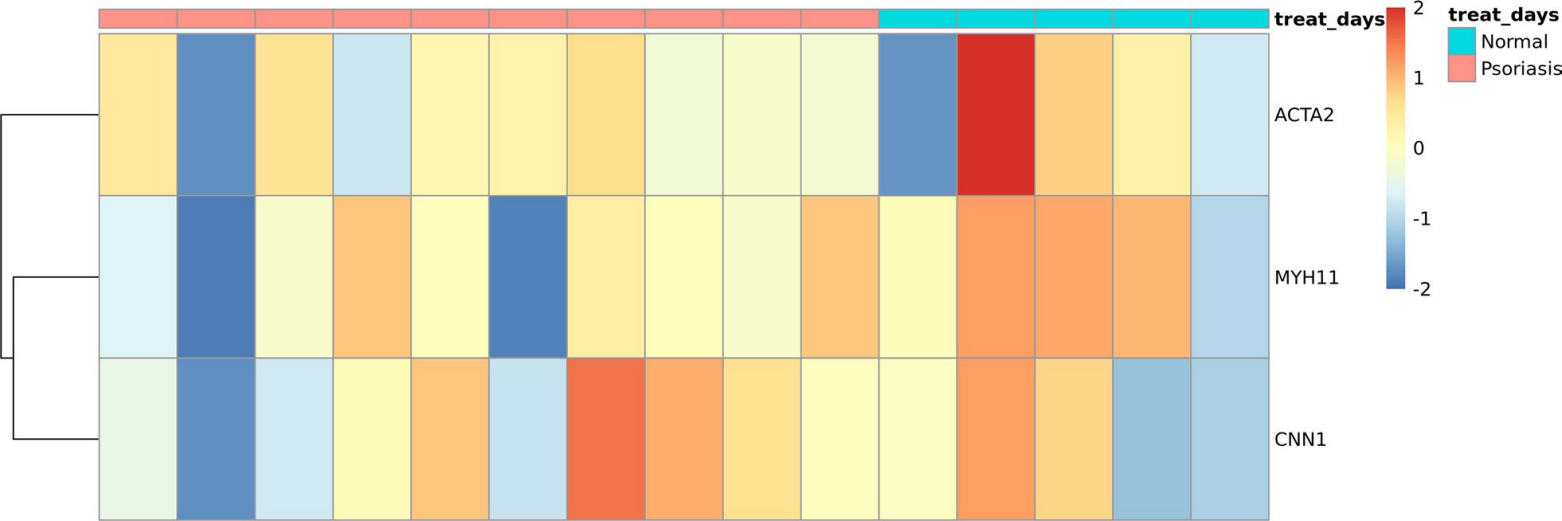
Down_regulated hub gene differential expression heatmap

The external validation for these hub genes was performed using data from the gse158448. It was observed that the hub gene has a similar expression pattern in the external dataset, which shows the confidence and the importance of our discovery and the therapeutic potential of the hub genes. ("Fig. 15 Up_regulated hub gene differential expression heatmap Fig. 16 Down_regulated hub gene differential expression heatmap).

## Discussion

Psoriasis is an inflammatory skin disease substantially diminishes patients’ quality of life and is associated with multiple comorbidities as well. The cornerstone for treating mild psoriasis is still remained topical therapies. But for moderate to severe plaque psoriasis, therapeutic advancements include biologics such as TNF-α, IL-12/23, IL-17, and IL-23, as well as an oral phosphodiesterase 4 inhibitor. The most common variant of psoriasis is plaque psoriasis. It is accounting for more than 80% of the psoriasis cases.

Plaque psoriasis is characterized by plaques or erythematous scaly patches that occur commonly on extensor surfaces. But it can also affect the palms, soles, intertriginous areas and nails. Psoriasis affects adults more than children, and it affects men and women equally [7, 29, 30]. Plaque psoriasis is an inflammatory immune-mediated skin disorder. And its approximate prevalence is 1–4% globally [31–33]. Interleukin (IL)-23 contributes to psoriasis by maintenance, stimulating proliferation, and differentiation of T-helper 17 cells and innate immune cells which produce proinflammatory cytokines such as IL-17 [34, 35]. Secukinumab, a human IgG1 monoclonal antibody which was approved in 2015 for the treatment of plaque psoriasis by targeting IL-17A, and has demonstrated greater efficacy than ustekinumab [19, 36]. The discovery of the IL-23/IL-17 immunologic pathway was a key to expand our knowledge about the pathogenesis of psoriasis and develop new targeted therapeutic agents about psoriasis as well [17, 37]. Although approved biologics, such as secukinumab are effective in the treating of plaque psoriasis, there is still a need for more efficacious therapy that will maintain and achieve higher response rates in the long term [38, 39]. This study dues to bioinformatics methods to find out the immune response genes of secukinumab, which provides important guiding significance for the action mechanism of unmask drugs and the development of more efficient drugs. During disease development, gene chip technology can reveal tens of thousands of genetic changes, it will provide promising therapeutic targets for diseases. IκBζ plays a crucial role in the antipsoriatic effects mediated by anti–IL-17A treatment. At the same time, blockade of IL-17A by secukinumab leads to clinical, histologic, and molecular resolution of psoriasis [40].

In this study, we validated the effect of the treatment with secukinumab in psoriasis through molecule. We analysis the significance involved in psoriasis-related biological functions and signaling pathways among the group of 1936 DEGs. We applied the ssGSEA methodology to recognize the immune cell infiltration related to the samples and identified hub genes that may be closely related to the treatment of psoriasis. We have analyzed the interaction network between them, signal transduction pathways and their biological functions.

Among the two groups of DEGs, we found 8 common genes, which may be closely related to the treatment of psoriasis with secukinumab. Interleukin-17 (IL-17) pathway, one of the most famous immune processes underlying the pathogenesis of psoriasis, showed a strong enrichment effected on epigenetic variation and psoriasis-related genes [41]. Moreover, IL-17A can promote the proliferation of epidermal keratinocytes for an imbalance between the proliferation and differentiation of keratinocytes in patients [42, 43]. The inhibitory effect of anti-IL-17A on psoriasis plays a significance role in the early histopathological, molecular and clinical treatment of psoriasis [44].

However, there are still some limitations in our study. We need generate the microarray data by the authors besides obtained from the GEO database. In addition, these target genes should be verified if those can be used in the clinical treatment of psoriasis through further experiments.

During the recent study, one of the most useful method to identify pathways is considered risk subpathway analysis based on DEGs which mostly related to psoriasis. Reasons for psoriasis was proved epidermal cell differentiation,keratinocyte differentiation and keratinization. Pathways, such as pyrimidine metabolism, folate biosynthesis and steroid hormone biosynthesis are the pathogeneses of psoriasis. In our study, PTG1, HMMR, PBK, FEN1, DEPDC1 may be some psoriasis-related genes in IL-17A treating of psoriasis. They are significant targets in psoriatic therapy. Our study provided probable target genes for psoriatic therapy through exploring secukinumab in psoriasis treatment.

## Data Availability

https://www.ncbi.nlm.nih.gov/geo/query/acc.cgi?acc=GSE137218. https://www.ncbi.nlm.nih.gov/geo/query/acc.cgi?acc=GSE158448.
